# Resveratrol for Cancer Treatment: Effects on Metabolism and Immune Cells

**DOI:** 10.3390/biom16010118

**Published:** 2026-01-09

**Authors:** Rongrong Bao, Tianrui Wang, Wenkai Jiang

**Affiliations:** 1 The First School of Clinical Medicine, Lanzhou University, Lanzhou 730000, China; 320220908830@lzu.edu.cn (R.B.); 320220909641@lzu.edu.cn (T.W.); 2 School of General Medicine, Xi’an Medical University, Xi’an 710077, China; 3 The First Clinical Medical School, Xi’an Medical University, Xi’an 710077, China

**Keywords:** resveratrol, cancer, metabolism, immune cells

## Abstract

Resveratrol is a natural polyphenol found in plants that has attracted significant research attention for its antitumor potential. With the continuing research on the tumor microenvironment and metabolic reprogramming, the roles of resveratrol in tumor cell metabolism and immune cell function have gained increasing attention. Recent studies have shown that resveratrol disrupts tumor cell metabolism and prevents tumor cell growth and metastasis by inhibiting metabolic processes such as glycolysis and fatty acid production. Furthermore, resveratrol regulates immune cells such as T cells, macrophages, and natural killer cells and enhances antitumor immune responses. In this article, we report the recent research progress on the use of resveratrol for tumor therapy. Specifically, we focus on its regulatory effect on tumor metabolism and the immune microenvironment and discuss its potential in combination with a new therapeutic strategy.

## 1. Introduction

Cancer is the second most common cause of death worldwide and is a major social, medical, and economic issue in the 21st century, placing a great burden on medical systems. According to the latest data, there are close to 19 million cancer cases and 10 million cancer deaths (excluding nonmelanoma skin cancers) worldwide. In the absence of intervention, these numbers are projected to increase to 33 million cancer cases and 18 million cancer deaths by 2050 [1]. Conventional treatments include mainly surgical excision, radiation therapy, and chemotherapy. However, these methods have significant limitations. In recent years, targeted therapy and immunotherapy have become important research fields due to their high specificity and efficiency [2]. The biological properties of cancer cells are closely related to metabolic reprogramming; thus, treatments targeting important metabolic molecules of cancer cells are attracting increasing attention [3,4].

Natural plant-based compounds are involved in many biological activities and have anti-inflammatory, antioxidant, antiaging, and antitumor effects. Resveratrol is a natural polyphenolic compound commonly found in the fruits and skins of plants such as grapes, blueberries, and pomegranates [5]. Studies have shown that resveratrol has a variety of biological effects, including antiaging, antitumor, anti-inflammatory, antioxidant, insulin-sensitizing, cardioprotective, and vasodilatory effects [6,7,8,9,10]. In particular, the anticancer effects of resveratrol and its mechanisms have been widely studied. Available evidence suggests that resveratrol can be used as a preventive and therapeutic drug for a variety of cancers, including breast, cervical, ovarian, skin, stomach, prostate, and liver cancer [11,12,13,14,15]. Increasing evidence indicates that resveratrol can regulate metabolic processes and immune functions. Given the significant roles of metabolic reprogramming and immune cell infiltration in the occurrence of tumors, further study of the effects of resveratrol on tumor metabolism and immune cells in the tumor microenvironment (TME) is necessary. This study focused on the regulatory effects of resveratrol on tumor metabolism and immune cells and analyzed the potential applications and underlying mechanisms of resveratrol in tumor treatment.

## 2. Chemical Properties and Biological Activities of Resveratrol

### 2.1. Chemical Structure and Sources

**Figure 1:**
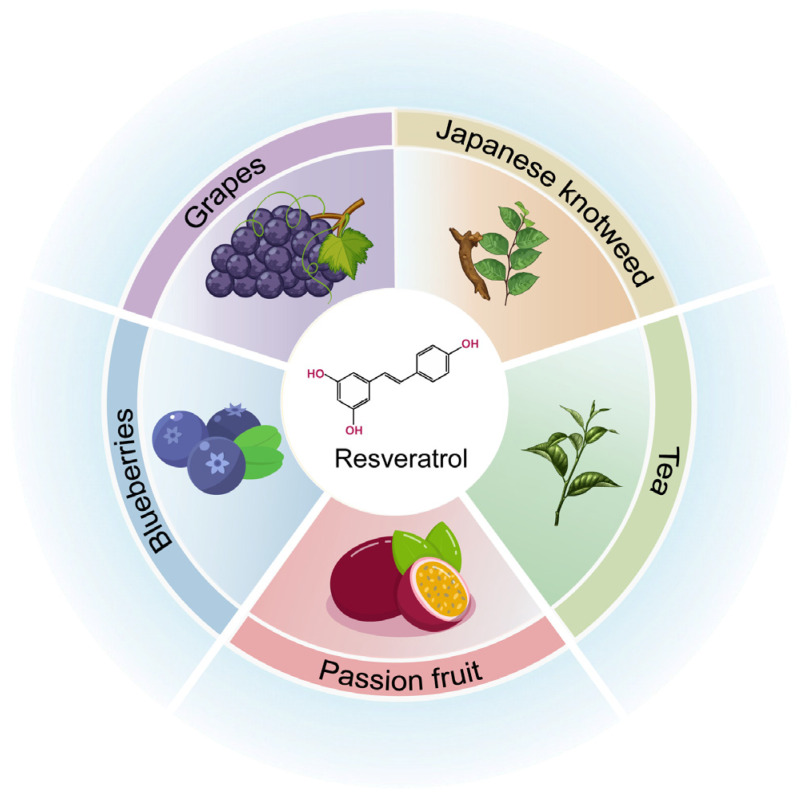
Chemical structure and sources of resveratrol.

Resveratrol (3,5,4-trihydroxydiphenyl ether) is a compound commonly found in plants and can be obtained from various food sources, such as grapes, passion fruit, tea, berries, pomegranate, almond, red wine, and Japanese fern (*Polygonum cuspidatum*) [16] (Figure 1). Structurally, resveratrol is formed by connecting two aromatic rings through a methylene bridge and exists in both cis and trans isomers. Most of its biological activities are generated through conversion processes; although it is stable under acidic and ambient conditions, it rapidly degrades in alkaline environments. The stability of liquid formulations can be improved by controlling the pH, temperature, oxygen, and light [16,17]. In industrial production, resveratrol is synthesized mainly through the Heck reaction catalyzed by palladium. The substrates of this reaction are aryl precursors (such as aryl halides or aryl carboxylic acid derivatives) and styrene derivatives (such as 4-acetoxy styrene). This reaction has a high yield, the catalyst can be reused, and it has great potential for large-scale production [18].

### 2.2. Bioavailability and Pharmacokinetics

The oral bioavailability of resveratrol is very limited, generally less than 1%, which is largely attributed to its rapid metabolism and pronounced first-pass effects. This compound undergoes glucuronidation and sulfation in the intestines and liver, which convert it into biologically inactive metabolites such as resveratrol-3-O-glucuronide and sulfates. Approximately 70% is ultimately excreted from the body via feces and urine [19]. Following oral intake, resveratrol is rapidly absorbed and reaches maximum levels in blood plasma within 1.5–2 h under conditions without food intake [20]. Moreover, the half-life of this compound is short, ranging from 1.90 to 1.23 h in mice, which further suggests that elimination occurs rapidly [21].

Various approaches have been developed to improve bioavailability. These include delivery systems using very small carriers such as liposomes and polymeric micelles [22,23], structural modifications such as covalent coupling with aspirin to generate resveratrol-aspirin derivatives [24,25], and local sustained-release systems such as polylactic glycolic acid (PLGA) scaffolds [26]. For example, placing PLGA scaffolds into fat tissue can reduce how often the compound is administered and improve the local processing of fats by activating adenosine monophosphate-activated protein kinase (AMPK) signaling [27]. In addition, the affinity of resveratrol for fats allows it to cross the blood–brain barrier efficiently, which appears critical for providing protection to nerve tissue [28].

Collectively, these delivery systems (e.g., liposomes, PLGA, and carbon dots) provide promising strategies to overcome the inherent pharmacokinetic limitations of resveratrol.

### 2.3. Biological Activities

Resveratrol has multiple effects on the body, including antioxidant, anti-inflammatory, cardioprotective, and immunomodulatory effects [29]. It exerts antioxidant effects not only by directly scavenging reactive oxygen species but also by activating the nuclear factor erythroid 2-related factor 2 (Nrf2) signaling pathway to increase the expression of antioxidant enzymes such as superoxide dismutase and glutathione peroxidase [30]. Moreover, it has particular anti-inflammatory effects due to its ability to inhibit the nuclear factor-kappa-light-chain-enhancer of activated B cells (NF-κB) pathway. It inhibits the activation of inhibitor of kappa B (IκB) kinase and the nuclear diffusion of NF-κB and downregulates the expression of proinflammatory cytokines such as tumor necrosis factor alpha (TNF-α) and interleukin-6 (IL-6) [31].

The main mechanism of resveratrol includes metabolic regulation in different disease contexts. It regulates cellular metabolism by affecting glucose uptake and utilization as well as glycolytic pathways and mitochondrial function [32]. By activating the sirtuin 1 (SIRT1)/AMPK pathway, resveratrol restores energy homeostasis and reduces lipid accumulation [33]. Furthermore, it regulates key metabolic players such as the glucose transporter GLUT-4 and the adipokine leptin, contributing to its active role in obesity caused by insulin resistance and metabolic disorders [34].

Through its effects on various immune cell populations, resveratrol strongly regulates the immune system. It regulates the SIRT1–NF-κB axis, increases the production of antitumor cytokines, and reshapes immune responses in various inflammatory environments [35]. This compound encourages macrophages to polarize toward an anti-inflammatory M2 phenotype and increases CD8^+^ T cell and natural killer (NK) cell activity in autoimmune diseases and chronic inflammation [36]. By inhibiting the janus kinase 2/signal transducer and activator of transcription 3 (JAK2/STAT3) signaling, resveratrol can also regulate T cell infiltration and function in inflammatory diseases [37]. Moreover, its interaction with B-cell lymphoma 6 influences B-cell activity in immune-mediated diseases [38].

Resveratrol has unique value in the prevention and treatment of chronic diseases due to its interactive regulation of metabolism and the immune system. The compound can inhibit the toll-like receptor 4 (TLR4)/NF-κB signal transduction process and subsequently reduce the production of proinflammatory cytokines such as IL-6 and TNF-α [39,40]. Moreover, resveratrol can improve metabolic function, increase mitochondrial efficiency, and maintain energy homeostasis by activating the SIRT1 and AMPK signaling pathways. This effect is reflected in the increase in adenosine triphosphate (ATP) content in the hippocampus in neurodegenerative disease-related models [40,41]. Resveratrol can also regulate the composition of the intestinal flora, increase the number of beneficial microorganisms, and reduce the proportion of Gram-negative bacteria; a reduction in the number of Gram-negative bacteria can reduce the level of lipopolysaccharide in the circulation, thereby reducing the systemic inflammatory response [39,42]. By relying on the above multiple mechanisms of action, resveratrol can help rebuild the balance between immunity and metabolism, which suggests its therapeutic potential for diabetes, neurodegenerative diseases, cancer and other diseases [41,42,43].

Collectively, these diverse biological activities are mediated through a complex network of signaling pathways. To provide a comprehensive overview, the key molecular targets and underlying mechanisms of resveratrol in cancer therapy are summarized in Table 1.

### 2.4. Pro-Apoptotic Activity in Cancer

#### 2.4.1. Overview

Apoptosis is a crucial programmed cell death mechanism for tissue homeostasis and is commonly dysregulated in cancer [97]. Cancer cells evade death by upregulating antiapoptotic proteins (e.g., the B-cell lymphoma 2 (Bcl-2) family, survivin, and X-linked inhibitor of apoptosis protein (XIAP)) and inhibiting proapoptotic complexes (e.g., apoptosomes and FADDosomes) [98,99,100,101,102]. Resveratrol functions as a multi-target modulator to reactivate these apoptotic pathways, demonstrating significant therapeutic potential.

#### 2.4.2. Suppression of Anti-Apoptotic Mechanisms

Resveratrol overcomes resistance to apoptosis by inhibiting key survival signaling pathways. It blocks PI3K/Akt and NF-κB signaling, thereby reducing the transcription of downstream antiapoptotic proteins [85,86,87,88]. Specifically, resveratrol disrupts the balance of the Bcl-2 family by downregulating Bcl-2 and upregulating Bcl-2-associated X protein (Bax), leading to an increased Bax/Bcl-2 ratio. This change triggers mitochondrial membrane depolarization and cytochrome c release [88,89,90]. Furthermore, resveratrol degrades “inhibitor of apoptosis proteins” such as survivin and XIAP. This removes the suppression of caspases, facilitating the activation of the caspase cascade and subsequent cell death [92,93,99,103].

#### 2.4.3. Activation of Pro-Apoptotic Pathways

Resveratrol simultaneously triggers both extrinsic and intrinsic apoptotic pathways and enhances their cross-talk. In the extrinsic pathway, it promotes Fas receptor aggregation and FADDosome assembly, leading to caspase-8 activation [94].

In the intrinsic pathway, it promotes the formation of the apoptosome by upregulating apoptotic protease activating factor 1 (Apaf-1) and inducing cytochrome c release, which activates caspase-9 and downstream executioner caspases [90,91,104,105].

**Figure 2:**
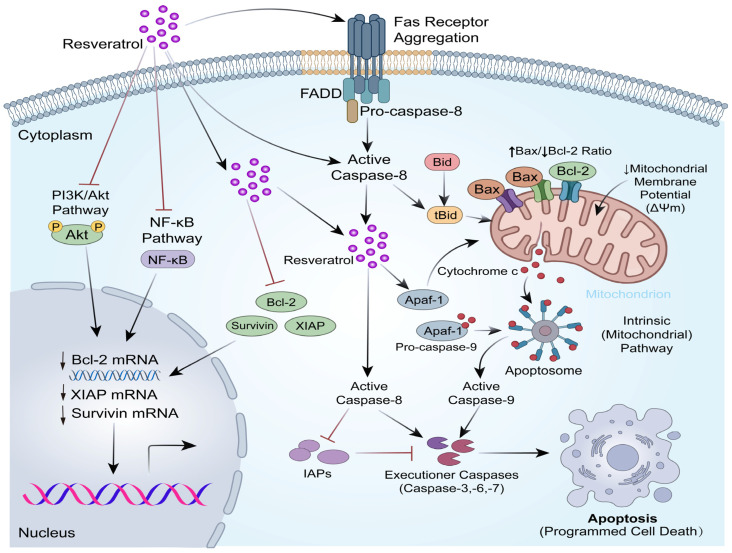
Resveratrol-mediated regulation of apoptosis in cancer cells. The figure illustrates that resveratrol exerts its anticancer effects by up-regulating pro-apoptotic targets (such as the Apoptosome, FADDosome, and Caspases) and down-regulating anti-apoptotic proteins (such as Bcl-2, Survivin, and XIAP). The red arrows in the figure represent inhibition, while the black arrows represent promotion.

Crucially, resveratrol amplifies these signals through the BH3-interacting domain death agonist (Bid) protein. Activated caspase-8 cleaves Bid into truncated-Bid, which migrates to mitochondria to further stimulate cytochrome c release, creating a self-reinforcing loop that drives irreversible apoptosis [96,106] (Figure 2).

## 3. Effects of Resveratrol on Tumor Cell Metabolism

### 3.1. Regulation of the Glycolytic Pathway

Even under oxygen-rich conditions, cancer cells still convert glucose into lactic acid via fast glycolysis instead of the faster mitochondrial oxidative phosphorylation pathway—a phenomenon known as the Warburg effect. This is associated with signaling pathway mutations (such as aberrant activation of phosphoinositide 3-kinase/protein kinase B/mechanistic target of rapamycin (PI3K/Akt/mTOR) signaling), mitochondrial dysfunction, and reprogramming of the TME. This metabolic reprogramming not only provides abundant biosynthetic precursors for the rapid proliferation of cancer cells but also is an important target for tumor diagnosis and treatment [3,107,108].

Resveratrol exhibits antitumor properties through the targeting of numerous steps in the process of glycolysis and significantly suppresses cancer metabolism. Resveratrol can directly inhibit the expression of the glucose transporters GLUT1, hexokinase 2 (HK2), lactate dehydrogenase A (LDHA), and phosphofructokinase (PFK), blocking glucose uptake and lactate production [44,45,47]. In addition, resveratrol can disrupt the Warburg effect, induce autophagy by activating AMPK/mTOR signaling, and reduce oxidative stress and insulin resistance in liver cancer [45,46]. In ovarian cancer studies, it reversed the EMT triggered by IL-6, inhibiting the migration and invasion of tumor cells, with synergistic antitumor effects together with the glycolytic inhibitor analog 2-deoxy-D-glucose [32].

### 3.2. Mitochondrial Function and Oxidative Phosphorylation

Although tumor cells favor aerobic glycolysis (the Warburg effect), their mitochondrial oxidative phosphorylation (OXPHOS) function persists through complex metabolic reprogramming. Mitochondria support tumor cell proliferation, survival, and microenvironmental adaptation by maintaining the bioenergy supply, providing biosynthetic precursors, regulating reactive oxygen species (ROS) signaling, and enhancing antiapoptotic capacity [109,110,111].

Resveratrol exerts anticancer effects by targeting multiple mitochondrial processes to interfere with metabolism and induce cell death. It directly inhibits complexes II and III of the electron transport chain (ETC) [48] and impairs OXPHOS, reducing ATP production. This disruption decreases the mitochondrial membrane potential (ΔΨm). The dissipation of the ΔΨm triggers opening of 1-methyl-4-phenyl-1,2,3,6-tetrahydropyridine (mPTP). Consequently, this causes the release of cytochrome c into the cytosol, which in turn activates caspase-dependent apoptosis [49,51] and results in severe oxidative damage and increased apoptotic signaling. Moreover, resveratrol treatment promotes the accumulation of mitochondria-derived reactive oxygen species (mtROS) [49,51], leading to severe oxidative damage and heightened apoptotic signaling. Specifically, a burst of mtROS can activate TXNIP/NLRP3 inflammasome signaling and pyroptosis [50]. Additionally, resveratrol blocks mitochondrial biogenesis through the AMPK/SIRT1/PGC-1α axis [52], inhibits the activity of glycolytic enzymes (HK2) through the PI3K/Akt/mTOR signaling pathway [52,112], and enables tumor cells to escape aerobic glycolysis. The combination of resveratrol and chemotherapeutic drugs such as 5-fluorouracil can reduce ATP levels, increase ROS production, increase mitochondrial membrane depolarization, and enhance antitumor efficacy [113]. Mitochondrial-targeted delivery systems (such as resveratrol carbon dots) can specifically enrich mitochondria and destroy mitochondrial function [48].

### 3.3. Reprogramming of Lipid Metabolism

In addition to the abnormalities in glucose metabolism, there are considerable differences in lipid metabolism between tumors and normal tissues, not only in the tendency of cancer cells to remodel lipid metabolism by increasing their own synthesis but also in the overexpression of key enzymes such as acetyl-CoA carboxylase (ACC), ATP citrate lyase (ACLY), and fatty acid synthase (FASN), which is also reflected in the process of enhanced lipid uptake from the environment through the cluster of differentiation 36 (CD36) pathway; these metabolic alterations are involved in important aspects of tumor cell biology. These processes include the synthesis of cell membranes, the formation of structures containing lipid droplets, β-oxidation processes for energy generation, and the production of signaling factors such as sphingosine-1-phosphate and lysophosphatidic acid [114,115,116].

Resveratrol has been shown to target different aspects of lipid metabolic reprogramming to achieve antitumor effects. The key mechanism involves the activation of SIRT1 and peroxisome proliferator-activated receptor alpha (PPAR-α), which promote fatty acid β-oxidation. Afterward, lipids are guided to the path of energy production rather than biosynthesis [53,54,55,56]. Moreover, resveratrol inhibits lipid synthesis in breast cancer cells, such as SK-BR-3, MCF-7 and MDA-MB-231 cells, both in vitro and in vivo. It can also inhibit the activity and expression of fatty acid synthase (FASN) to reduce lipid production, destroy membrane integrity and signal transduction processes, and induce apoptosis [57]. In addition, resveratrol can block the transport of fatty acids such as linoleic acid to the nucleus and the subsequent prometastatic gene expression mediated by PPARβ/δ through competitive binding to fatty acid binding protein 5 (FABP5), thus effectively inhibiting the migration and invasion of cervical cancer cells [13].

## 4. Regulatory Effects of Resveratrol on Immune Cells

### 4.1. Effects on T Cell Function

As the core component of adaptive immunity, T cells play a key role in the cellular immune response. Based on the differences in surface markers and functions, T cells can be divided into different subsets, among which CD4^+^ and CD8^+^ T cells are particularly important [117]. CD8^+^ T cells are the core effectors of antitumor immunity and can directly recognize and eliminate tumor cells. The core function of CD4^+^ T cells is to transmit costimulatory signals, which can not only increase the functional activity of CD8^+^ T cells but also contribute to the formation of memory T cells, thereby regulating the immune process [118,119]. In the TME, continuous antigen exposure combined with the action of inflammatory cytokines often leads to the exhaustion of CD8^+^ T cells, which promotes immune escape and tumor progression. Therefore, an immune intervention strategy designed for CD8^+^ T cells has important research value [118,120].

**Figure 3:**
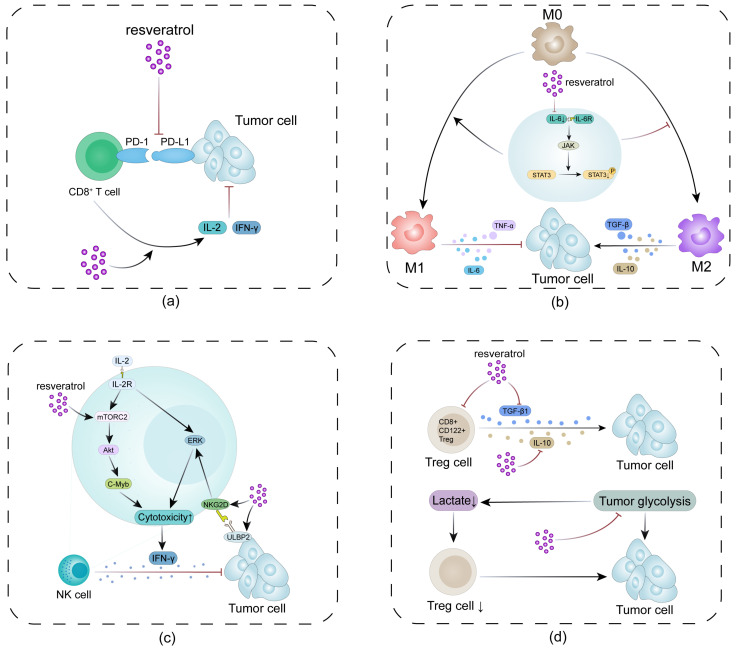
Molecular mechanisms underlying the regulatory effects of resveratrol on immune cells. (**a**) Resveratrol restores CD8^+^ T cell antitumor immunity via blockade of the PD-1/PD-L1 checkpoint axis; (**b**) resveratrol modulates macrophage polarization toward an antitumor M1 phenotype via inhibition of the IL-6/JAK/STAT3 signaling pathway; (**c**) resveratrol augments NK cell cytotoxicity and IFN-γ secretion through the mTORC2/Akt/c-Myb signaling cascade; (**d**) resveratrol attenuates Treg-mediated immunosuppression by targeting tumor glycolysis and reducing lactate accumulation. The red arrows in the figure represent inhibition, while the black arrows represent promotion.

Resveratrol has the potential to regulate the function of T cells in the immune microenvironment. It can promote the release of interferon gamma (IFN-γ), interleukin-2 (IL-2), and other effector cytokines; increase the expression level of CD107a; and significantly increase the killing activity and antitumor effect of CD8^+^ T cells [58,59,60]. Related studies have confirmed that it can reduce the expression of the immune checkpoint protein programmed cell death protein 1 (PD-1) on the surface of T cells, alleviate the exhaustion of T cells, and reconstruct the tumor-specific immune response [61,62,63,64]. In an experimental model of metastatic triple-negative breast cancer, resveratrol intervention significantly reduced PD-1 expression in CD8^+^ and CD4^+^ T cells in lung tissue, improved T cell exhaustion, enhanced cytotoxic function, promoted the Th1 cytokine response, and ultimately effectively inhibited tumor metastasis to the lung [59] (Figure 3a).

### 4.2. Regulation of Macrophage Polarization

Macrophages constitute the core component of the innate immune system; are widely distributed in various tissues, body cavities, and mucosal surfaces; and play key roles in the immune process of host defense against pathogen invasion and tumor defense [121]. Tumor-associated macrophages (TAMs) in the TME are relatively common infiltrating immune cells [122]. Their biological behavior directly affects tumor proliferation, angiogenesis, and therapeutic tolerance potential. These cells present two main polarization phenotypes: M1 macrophages, which have both proinflammatory properties and antitumor activity, and M2 macrophages, which have anti-inflammatory characteristics and can promote tumor progression [123,124]. The dynamic regulatory relationship between these two factors is indispensable for maintaining tumor microenvironment homeostasis.

Resveratrol can regulate macrophage polarization to remodel the internal immune environment of tumors. Studies have confirmed that it can promote the polarization of macrophages to the M1 phenotype with proinflammatory characteristics, improve antigen-presenting function, promote the release of inflammatory cytokines such as TNF-α and IL-12, and inhibit the activation of M2 macrophages with protumor effects, thus enhancing the immune response capacity of the body [125,126,127]. This immunomodulatory effect depends mainly on the activation of the STAT3 pathway, which effectively inhibits M2 polarization-related signals by affecting cell metabolism and epigenetic regulatory mechanisms [65]. For example, in a breast cancer model, resveratrol promoted M1 polarization while blocking IL-6/STAT3 signaling, inhibited M2 polarization, improved the TME, and increased tumor sensitivity to chemotherapy [66]. Resveratrol can also reduce the release of inflammatory factors from macrophages by inhibiting the NF-κB signaling pathway, suggesting that it may have application value in the treatment of autoimmune diseases and chronic inflammation [67,68]. These results reveal the multidimensional regulation of macrophage polarization, providing a basis for its potential application in the field of tumor immunotherapy (Figure 3b).

### 4.3. Increased NK Cell Activity

NK cells originate from bone marrow precursors and play a key role in the body’s tumor defense process as the core component of innate immunity [128,129]. These cells can directly recognize and clear tumor cells through a variety of mechanisms: the secretion of perforin and granzyme triggers the programmed death of target cells, initiates the apoptosis signaling pathway with the help of the Fas/FasL receptor interaction, and recognizes antibody-labeled tumor cells through the CD16a receptor to mediate antibody-dependent cytotoxic effects [130,131]. In addition to their direct killing efficacy, NK cells can also secrete a variety of cytokines, which can activate dendritic cells, enhance T cell response activity, inhibit immunosuppressive cells, and change the immune status of the TME as a whole to play an indirect antitumor role [132].

Studies have confirmed that resveratrol can effectively enhance the antitumor efficacy of NK cells [70], and animal experiments have shown that it can increase the level of IFN-γ, increase the cytotoxicity of natural killer cells, and significantly inhibit tumor proliferation and diffusion [133]. At the mechanistic level, resveratrol combined with IL-2 can more efficiently activate NK cells through the two core signaling pathways, natural killer group 2, member D/mitogen-activated protein kinase (NKG2D/MAPK) and mechanistic target of rapamycin complex 2/protein kinase B/c-Myb (mTORC2/Akt/c-Myb). Combined treatment promotes the secretion of perforin and IFN-γ and further enhances the ability of NK cells to clear tumor cells [69,70]. Studies related to breast cancer models further revealed that resveratrol could downregulate the expression of the microRNA miR-17-5p, relieve its inhibitory effect on misshapen-like kinase 1 (MINK1), and thus activate the downstream MINIK1/c-Jun N-terminal *protein* kinase/c-Jun (MINK1/JNK/c-Jun) signaling axis. This process significantly enhances the expression of the tumor cell surface ligand ulbp2 so that NK cells can more efficiently recognize and kill target cells through the NKG2D receptor. The above findings have been verified in cell experiments and animal models, highlighting the potential application value of resveratrol in the field of tumor therapy [71].

Moreover, resveratrol can strengthen the ability of chimeric antigen receptor natural killer (CAR-NK) cells to resist oxidative stress. Its mechanism of action is reflected in its ability to activate the intracellular antioxidant defense system, reduce the accumulation of reactive oxygen species, and upregulate the expression of multiple antioxidant-related genes. Moreover, the compound can optimize cellular energy metabolism, improving the efficiency of mitochondrial oxidative phosphorylation while accelerating the glycolysis process. These changes enhance both the metabolic adaptability and the tumor-killing function of CAR-NK cells [72,134]. As a natural immune regulator, resveratrol has important application prospects in the field of NK cell-based transplantation immunotherapy (Figure 3c).

### 4.4. Suppression of Regulatory T Cells

Resveratrol can also suppress regulatory T cells (Tregs), a key population of immunosuppressive cells in the TME, which are pivotal for suppressing antitumor immunity and facilitating tumor immune escape [135]. In a mouse model of hepatocellular carcinoma, the proportions of immunosuppressive CD8^+^CD122^+^ Tregs in the tumor interior and peripheral lymphoid organs were effectively reduced after resveratrol intervention, and the transplantation of exogenous CD8^+^CD122^+^ Tregs partially reversed the antitumor effect of resveratrol, confirming that such cells are the key targets through which they play a role [73], whereas the levels of the immunosuppressive cytokines IL-10 and TGF-β1 in the TME decreased synchronously [73].

Resveratrol can also indirectly affect the function of Tregs by altering the metabolic characteristics of the TME. It can impede the tumor glycolytic process and reduce the production of lactic acid, which is an important metabolite for maintaining the activity and stability of Tregs. Studies have shown that resveratrol treatment via a codelivery system can achieve metabolic reprogramming and reduce the lactate concentration, which is closely related to the reduction in invasive Tregs in tumors and the remission of the immunosuppressive state [74]. Resveratrol antagonizes the immunosuppressive effect mediated by Tregs through the dual pathways of direct inhibition of cell function and indirect regulation of metabolism (Figure 3d).

### 4.5. Impact on MDSCs

Myeloid-derived suppressor cells (MDSCs) belong to the immature myeloid cell population and have obvious heterogeneity. They can be recruited in large numbers to the TME and drive tumor progression by inhibiting the immune response, promoting angiogenesis, and accelerating metastasis. This cell population mainly includes granulocytic MDSCs (G-MDSCs) and monocytic MDSCs (M-MDSCs) [136], which are closely related to the occurrence of primary and secondary drug resistance in cancer immunotherapy [137].

Resveratrol has potential therapeutic value through the regulation of MDSCs. Studies have shown that it can reduce the number of granulocytic MDSCs in tumor tissue and the spleen in two ways: triggering apoptosis while blocking the recruitment process via the inhibition of tumor-derived high-mobility group box 1 (HMGB1). Moreover, this substance can downregulate arginase-1 levels and reduce reactive oxygen species production, alleviating G-MDSC-mediated immunosuppression to help restore the antitumor function of CD8^+^ T cells [75]. These results confirm that resveratrol plays multiple roles in relieving MDSC-mediated immunosuppression.

In particular, resveratrol has different effects on different subpopulations of MDSCs. The inhibition of G-MDSCs can promote the differentiation of M-MDSCs into mature CD11c^+^ and F4/80+ myeloid cells. This bidirectional regulatory effect suggests that it can be used as an adjunct to cancer immunotherapy by changing the status of immunosuppressive MDSCs and shortening the culture cycle of immunocompetent cells [75]. The above findings also provide a theoretical basis for the development of immunotherapy strategies against MDSCs.

## 5. Immunometabolic Reprogramming of T Cell Function

There is a common nutrient shortage in the TME, and saturated T cells need to deal with intense metabolic competition. Tumor cells use a high sugar fermentation rate (Warburg effect) to quickly consume local glucose, and lactic acid accumulates, directly reducing the toxicity of CD8^+^ T cells [138,139]. The metabolic disorders of T cells gradually fall into the “exhaustion” state, function continues to decline, and mitochondrial function abnormalities, bioenergy generation reduction and inhibitory receptor stabilization occur [140]. Improving mitochondrial adaptability and enhancing metabolic flexibility has become the core strategy for rebuilding antitumor immunity.

Resveratrol is an efficient immunometabolic regulator that can overcome the limits of the TME by reprogramming T cell metabolism. The mechanism involves the activation of SIRT1 deacetylase, which activates the PGC-1α signaling pathway, leading to mitochondrial biosynthesis [141,142]. This activation process drives CD8^+^ T cell metabolism, from glycolysis to OXPHOS and fatty acid (FAOX), to maintain survival and increase potential in a low-glucose environment [141,143]. SIRT1-mediated metabolic reprogramming can also promote the longevity of central memory T cells and prevent the end of the cycle. Resveratrol is also targeted by immunologic detection point metabolism, and by inhibiting the glycosylase of PD-L1 maturation, it promotes its degradation and prevents T cell function from being inhibited [144].

The effect of resveratrol on the immune metabolism of T cells is characterized by a biphasic response. The effect pattern can be promoted or inhibited with dose changes [145]. On the one hand, at physiological concentrations (e.g., <20 µM), this compound mainly supports immune regulation and can precisely control the cytokine profile of human T cells [146]. On the other hand, the higher pharmacological dose (typically >50 µM) inhibits the activation and proliferation of T cells and is related to key metabolic pathways, such as the blockade of mTOR [147]. This narrow treatment window suggests that its precise dose is a potential application for immunometabolic conditioners.

## 6. Resveratrol Remodels the Tumor Microenvironment via Metabolic–Immune Cross-Talk

Research has confirmed that there is a close relationship between the metabolic reprogramming of tumor cells and the formation of an immunosuppressive TME [148]. Resveratrol can simultaneously affect metabolism and immunity and regulate their interaction to achieve dual regulatory effects. It can destroy the immunosuppressive tumor microenvironment, providing a new direction for combined immunotherapy strategies.

Tumor cells are highly dependent on aerobic glycolysis, which leads to massive accumulation of lactic acid, in turn triggering acidification of the TME. An acidic environment inhibits the activity of p38 and the JNK/c-Jun signaling pathway and significantly reduces the killing abilities of T cells and NK cells. The metabolic characteristics of glucose deprivation and lactate accumulation also promote the activation of Tregs to further enhance the immunosuppressive effects. Lactate can also induce TAMs to polarize toward the tumor-promoting M2 phenotype. Taken together, these findings suggest that lactate builds an immunosuppressive niche conducive to tumor survival and proliferation through the remodeling of the TME [149].

Resveratrol can inhibit the glycolytic metabolism process and reduce lactic acid accumulation in TME, thereby weakening the immunosuppressive function of Treg cells. A study of an ID8 homologous ovarian cancer mouse model revealed that after resveratrol intervention, the expression levels of the key glycolytic enzymes PKM2 and GLUT1 are strongly regulated, and the lactate concentration in the tumor interstitial fluid is significantly reduced. These metabolic changes not only inhibit the proliferation and function of infiltrating Tregs in tumors but also promote the expansion of CD4^+^ and CD8^+^ T cell subsets that can produce TNF-α. These results demonstrated that reducing the level of lactate could effectively relieve the immunosuppression mediated by Tregs while activating antitumor immune responses [76]. Resveratrol achieves metabolic reprogramming of the TME by regulating lactate metabolism, which not only reverses the inhibitory effect triggered by Tregs but also enhances antitumor immune activity.

Resveratrol can also restore the cytotoxic function of T cells and NK cells by regulating lactate metabolism. Acidosis triggered by lactate inhibits the p38 and JNK/c-Jun pathways and subsequently weakens the activation and effector functions of these two immune cells. Resveratrol can specifically reduce the expression levels of key glycolytic enzymes such as PKM2 and LDHA, reduce glucose uptake and lactate production, and directly increase acidosis and hypoxia in the TME. After the lactate level decreases, the functional inhibition of cytotoxic T lymphocytes and NK cells is also alleviated [77]. Studies have confirmed that the topical application of resveratrol can inhibit the expression of PKM2 and LDHA, significantly reduce the glycolysis rate and lactate production, and reduce the degree of acidification of the TME. This metabolic reprogramming directly reverses the inhibitory effect of lactate on T cell proliferation and effector function while improving the energy metabolism of immune cells [77].

Resveratrol can also regulate the phenotypic polarization of TAMs by affecting lactate metabolism. The specific mechanism involves the inhibition of glycolysis and lactate production, thereby blocking the transformation of macrophages to the M2 type. A reduction in M2 polarization is helpful for relieving immunosuppression and improving the antitumor ability of the body. This mechanism has been validated in not only tumor models but also studies of inflammatory diseases such as psoriasis. In these models, resveratrol effectively alleviates the inflammatory response and immune cell infiltration by blocking the glycolytic process and lactate release in macrophages [150]. These results suggest that reprogramming the functions of macrophages associated with the glycolytic lactate axis is among the core mechanisms through which resveratrol exerts its immunomodulatory effects.

## 7. Limitations and Future Research Direction

### 7.1. Limitations of Resveratrol in Cancer Therapy

Resveratrol has shown multidimensional antitumor activity in preclinical research; however, its clinical translation has encountered significant obstacles, making its use as a conventional anticancer drug difficult. The core limiting factors are poor pharmacokinetic characteristics and low bioavailability. After oral administration, the compound undergoes rapid first-pass metabolism. In addition, its solubility in aqueous and lipid environments is not ideal. Its oral bioavailability is usually less than 1%, and its systemic absorption rate is only approximately 0.5% [151,152]. This characteristic prevents most of the administered dose from entering the systemic circulation and reaching the target tissue. Even if a small amount is absorbed, the short half-life also makes it difficult to maintain a sufficient therapeutic concentration in the body; a concentration above 25 μM is usually required to induce cancer cell death. To solve this problem, researchers have explored a variety of solutions, including the use of new delivery systems such as liposomes, nanoparticles, or hydrogels; the preparation of methylated derivatives (such as resveratrol methyl ester); or the combination with metabolic inhibitors. The development of resveratrol carbon dots in 2022 is a typical case. This technology significantly improves the drug uptake efficiency and mitochondrial targeting ability of cells, and the anticancer effect is better than that of ordinary resveratrol [48].

Several clinical trials have evaluated the safety and efficacy of resveratrol in patients with cancer, with variable results (Table 2). Positive biological activities, such as modulation of tumor biomarkers (e.g., activated caspase-3), have been observed in some studies and are well tolerated [153]; In others, no substantial clinical response was observed or safety concerns were revealed. A phase 2 trial in multiple myeloma, for example, was stopped early because of severe nephrotoxicity, a finding that highlights potential risks in specific patient populations [154]. Table 2 summarizes the results of representative clinical trials to demonstrate both the potential value of preclinical research findings to clinical translation and the main obstacles in this process.

Despite promising preclinical data, the transformation of existing achievements into clinical practice faces many obstacles. The potential toxicity and safety characteristics of resveratrol have not been fully defined, and this problem is more prominent when resveratrol is used at high doses or for a long time. A clinical trial for multiple myeloma has reported nephrotoxic reactions that need attention. Moreover, its interactions with other drugs also raise concerns, especially when resveratrol is used in combination with conventional chemotherapy drugs or metformin. As a phytoestrogen, resveratrol has both estrogen mimetic and antagonistic effects and may have complex effects on hormone-dependent tumors such as breast cancer. Notably, its usual antioxidant capacity may be converted into pro-oxidant activity under specific conditions, thereby causing cell damage [159].

At present, the application of resveratrol in tumor treatment is still focused on basic research and small-scale clinical exploration, and the transformation of existing achievements into clinical practice faces many obstacles, which are due mainly to the complex biological properties of the compound and the lack of reliable clinical data; thus, its practical application in the medical field still faces major challenges.

### 7.2. Future Research Directions

Future research on resveratrol should focus on the development of combination therapies and the exploration of intervention strategies for the treatment of cancer. One of the core research directions is to combine resveratrol administration with conventional chemotherapy, targeted therapy, immune checkpoint inhibitors, and other existing treatments to improve antitumor effects synergistically. Previous studies have confirmed that resveratrol combined with 5-fluorouracil can more effectively inhibit colorectal cancer cell proliferation [160] and may also reduce chemotherapy-related toxicity and reverse drug resistance. The application of nanocarrier delivery systems can increase the aggregation level of resveratrol at tumor sites and its ability to inhibit glycolysis [111], indicating good prospects for optimizing its therapeutic performance. Subsequent clinical trials need to focus on identifying the best combination of resveratrol with existing therapies. Chronic inflammation is the key factor that induces cancer. The anti-inflammatory and antioxidant properties of resveratrol are expected to be used to block the malignant transformation of cells. An important future research topic will be to comprehensively evaluate its safety and potential value as a chemopreventive agent through animal experiments combined with large-scale population studies.

### 7.3. Rational Design of Resveratrol Derivatives for Immunometabolic Optimization

Natural resveratrol has limitations in terms of pharmacokinetics and pharmacodynamics, and specialized structural optimization is needed to overcome these limitations. The development of resveratrol derivatives (SAR) (e.g., structural optimization methods) is a reliable way to enhance efficacy, selectivity, and drug properties. In traditional drug chemistry research, analogs that have improved anti-inflammatory, antioxidant, and enzyme inhibition effects have been successfully prepared. This finding indicates that targeted modification of the core stilbene skeleton can result in compounds with better biological activity [161]. For example, specific substitutions can significantly affect the ability of derivatives to regulate key cellular processes, such as oxidative stress and autophagy, which are closely related to immune cell function [162]. However, the experimental synthesis and screening of derivatives require a significant number of resources, and there is a lack of a reasonable basis for targeting specific immunological pathways.

This challenge is gradually being addressed through the application of computer simulations and artificial intelligence (AI) related strategies. These computational methods can rank derivatives designed for specific immune metabolic endpoints in a reasonable priority order. By integrating a hybrid approach combining structure-based molecular docking, ligand-based quantitative structure–activity relationship (QSAR) models, and machine learning classification, it is possible to predict binding affinity efficiently with key regulatory targets such as SIRT1 and AMPK in T cell metabolism [163,164]. In addition to single-target analysis, network pharmacology and bioinformatics modeling can achieve a systems-level understanding. By analyzing multiomics data, these methods can be used to map the effects of resveratrol on complex immune metabolic interaction networks, identify new vulnerable nodes in pathways associated with T cell exhaustion or mitochondrial dysfunction, and provide support for targeted intervention [165]. This workflow has been further optimized: for the above priority targets, a high-throughput virtual screening of compound libraries is conducted, the binding stability is evaluated through molecular dynamics simulations, and then the pharmacokinetic applicability is predicted through a comprehensive ADMET (absorption, distribution, metabolism, excretion, toxicity) analysis [163,164].

This kind of integrated research combining computation and experiments aims to overcome the limitations of conventional cytotoxic effects. The new generation of resveratrol derivatives needs to be optimized specifically for certain immune metabolic endpoints, which include enhancing the mitochondrial OXPHOS capacity of tumor-infiltrating T cells, increasing the reserve respiratory potential, and reprogramming-ming metabolic pathways to avoid depletion-related patterns. This approach is supported by evidence: Resveratrol can enhance antitumor immunity through targeting mitochondrial adaptability [166] and can also improve the efficacy of immune checkpoint blockade therapy by regulating systemic factors such as the gut microbiota [62]. By combining the design of derivatives with these precise functional results, research can shift from the preparation of broad-spectrum active compounds to the development of targeted molecular tools that can restore antitumor immunity at the metabolic level.

## 8. Conclusions

Resveratrol, as a natural polyphenol, has a variety of antitumor effects, and its biological effects rely mainly on intervening in the metabolic reprogramming of tumor cells and regulating the TME. In terms of metabolic regulation, compounds can effectively block the process of glycolysis, interfere with the normal function of mitochondria, and regulate lipid metabolism. These pathways disrupt energy acquisition and biomolecular synthesis in tumor cells. At the level of immune regulation, resveratrol can increase the killing efficacies of CD8^+^ T cells and NK cells and can also induce macrophages to differentiate into the M1 phenotype, with antitumor effects. Resveratrol can also inhibit the function of Tregs and relieve the immunosuppression triggered by MDSCs. These effects can promote the transformation of “cold” tumors with low immunogenicity into “hot” tumors with active immunity and further strengthen the effectiveness of immunotherapy. In addition, resveratrol can also regulate T cell immunometabolism, which mainly focuses on enhancing mitochondrial adaptability and metabolic stability of T cells. The compound can also promote cancer cell apoptosis through multiple pathways. However, there are still significant obstacles in the clinical application of this compound, with its low oral bioavailability and complex dose–response relationship representing the core issues. Future resveratrol research should pursue two integrated paths: developing its utility in combination therapies and chemoprevention, aided by nanodelivery, and employing computational design to create derivatives that precisely target immunometabolic pathways. This shifts the focus from broad activity to specific molecular tools for restoring antitumor immunity.

## Figures and Tables

**Table 1:** Molecular targets and mechanisms of resveratrol in cancer therapy.

Category	Targets/Pathways Involved	Mechanisms of Action	Functional Outcome	Refs.
Metabolic Modulation				
Glycolysis Inhibition	GLUT1, HK2, PKM2, LDHA, PFK; AMPK/mTOR pathway	Inhibits key glycolytic enzymes and glucose transporters; Activates AMPK signaling, antagonizing the Warburg effect and induce autophagy.	Impedes energy supply, inhibits tumor proliferation and metastasis.	[44,45,46,47]
Mitochondrial Modulation	ETC complexes II/III; mPTP; mtROS; TXNIP/NLRP3; AMPK/SIRT1/PGC-1α; PI3K/Akt/mTOR	Impairs OXPHOS, collapses ΔΨm, and induces cytochrome c release-mediated apoptosis; Triggers mtROS accumulation and NLRP3 inflammasome activation; Suppresses mitochondrial biogenesis.	Induces apoptotic and pyroptotic cell death.	[48,49,50,51,52]
Lipid Metabolism Reprogramming	SIRT1, PPAR-α; FASN; FABP5; PPARβ/δ	Promotes fatty acid β-oxidation; Suppresses de novo lipogenesis by inhibiting FASN; Antagonizes FABP5 to block fatty acid nuclear transport and pro-metastatic signaling.	Inhibits biosynthetic pathways and cancer cell migration/invasion.	[13,53,54,55,56,57]
Immunoregulation				
T cell Function	CD107a; PD-1;	Enhances CD8^+^ T cell cytotoxicity (IFN-γ, IL-2, CD107a); Attenuates exhaustion by downregulating PD-1; Note: Biphasic effect may occur at high concentrations.	Reverses T cell exhaustion and enhances antitumor immunity.	[58,59,60,61,62,63,64]
Macrophage Polarization	SIRT1/AMPK; NF-κB; IL-6/STAT3	Promotes repolarization from pro-tumoral M2 to antitumoral M1 phenotype; Enhances antigen presentation and pro-inflammatory cytokine secretion; Inhibits NF-κB-mediated inflammation.	Reprograms the TME, enhances phagocytosis, and improves chemosensitivity.	[65,66,67,68]
NK Cell Activation	NKG2D/MAPKs; mTORC2/Akt/c-Myb; MINK1/JNK/c-Jun: ULBP2	Upregulate the activity of NK cells; Downregulates miR-17-5p to elevate ULBP2 ligand expression on tumor cells via MINK1/JNK.	Boosts NK cell recognition and killing of tumor cells; Enhances cytotoxicity of CAR-NK cells.	[69,70,71,72]
Tregs Suppression	CD8^+^CD122^+^ Tregs; Tumor glycolysis;	Reduce CD8^+^CD122^+^ Treg frequency, decrease IL-10 and TGF-β1 levels, inhibit tumor glycolysis, decrease lactate production, disrupt lactate-dependent Treg stability	Alleviate immunosuppression and enhances antitumor immunity	[73,74]
MDSC Modulation	HMGB1; Arginase-1; ROS; M-MDSC differentiation pathways	Reduces G-MDSC accumulation, inhibit HMGB1-mediated recruitment, induces programmed cell death, decreases Arg-1 and ROS levels, promotes M-MDSC differentiation into mature myeloid cells.	Alleviates MDSC-mediated immunosuppression and restores CD8^+^ T-cell anti-tumor activity.	[75]
Metabolic-Immune Crosstalk				
Lactate Metabolism	PKM2, LDHA	Inhibits tumor glycolysis, reducing lactate production and accumulation in the TME.	Reverses lactate-mediated suppression of CTL and NK cell function and inhibits Treg activity.	[76,77]
Another Biological activities				
Apoptosis Induction	caspase-3, -8, -9; COX-2; AR/Akt; PIM-1 kinase; Bcl-2; Bax; Survivin; XIAP; Apoptosome; FADDosome; PI3K/Akt; Apaf-1; Bid; tBid	Activates intrinsic/extrinsic apoptotic pathways; Downregulates anti-apoptotic proteins; Inhibits pro-survival kinase signaling.	Directly induces tumor cell death.	[78,79,80,81,82,83,84,85,86,87,88,89,90,91,92,93,94,95,96]
Metastasis Suppression	NF-κB; Akt/GSK-3β/Snail axis; BCL6 BTB domain	Inhibits EMT by suppressing transcription factors (e.g., Snail); Downregulates EMT-related proteins; Disrupts oncogenic driver BCL6 (e.g., in NHL).	Suppresses tumor invasion and metastasis.	[38,78,79,80,81,82,83]
Antioxidant and Anti-inflammatory	Nrf2; NF-κB	Scavenges ROS directly; Activates Nrf2 pathway to upregulate antioxidant enzymes (SOD, GPx); Inhibits NF-κB activation and subsequent pro-inflammatory cytokine production.	Alleviates oxidative stress and chronic inflammation, preventing a procarcinogenic microenvironment.	[30,31,68]

Refs., references; GLUT1, Glucose Transporter Type 1; HK2, Hexokinase 2; LDHA, Lactate Dehydrogenase A; AMPK, AMP-activated Protein Kinase; PFK, Phosphofructokinase; ETC, electron transport chain; mPTP, mitochondrial permeability transition pore; mtROS, mitochondrial reactive oxygen species; OXPHOS, oxidative phosphorylation; ΔΨm, mitochondrial membrane potential; FASN, fatty acid synthase; FABP5, fatty acid-binding protein 5; PIM-1, Proviral Integration site for Moloney murine leukemia virus-1; GSK-3β, Glycogen Synthase Kinase 3 Beta; CTL, cytotoxic T lymphocyte; Treg, regulatory T cell; EMT, epithelial–mesenchymal transition; NHL, non-Hodgkin’s lymphoma; ROS, reactive oxygen species; PGC-1α, Peroxisome Proliferator-Activated Receptor Gamma Coactivator 1-alpha; Nrf2, nuclear factor erythroid 2–related factor 2; SOD, superoxide dismutase; GPx, glutathione peroxidase; Bcl-2, B-Cell Lymphoma 2; Bax, Bcl-2-Associated X Protein; XIAP, X-linked Inhibitor of Apoptosis Protein; IAPs, Inhibitor of Apoptosis Proteins; Apaf-1, Apoptotic Protease-Activating Factor 1;Bid, BH3-Interacting Domain Death Agonist; tBid, Truncated Bid.

**Table 2:** Summary of key clinical trials of resveratrol in cancer patients and prevention settings.

Study Phase/Design	Population (*n*)	Intervention and Dose	Key Outcomes (Positive and Negative)	Reference
Phase I (Pilot)	Colorectal Cancer (*n* = 9)	0.5–1.0 g/day for 8 days (pre-surgery)	Positive: Reduced tumor cell proliferation (Ki-67 decreased by 5%). Negative: No significant histological regression observed.	[155]
Phase I	Colorectal Cancer (*n* = 20)	Micronized resveratrol (SRT501), 5.0 g/day	Positive: Increased cleaved caspase-3 (apoptosis) in malignant tissue by 39%. Negative: Mild gastrointestinal adverse events (diarrhea).	[153]
Phase II	Multiple Myeloma (*n* = 24)	SRT501 (5.0 g/day) + Bortezomib	Positive: None significant in this setting. Negative: Trial halted early due to severe nephrotoxicity (cast nephropathy) in 5 patients; minimal efficacy.	[154]
RCT (Double-blind)	Breast Cancer Risk (*n* = 39)	50 mg or 500 mg (bid) for 12 weeks	Positive: Decreased methylation of tumor suppressor gene RASSF-1α. Negative: No significant effect on other genes or systemic estradiol levels.	[156]
Phase I	Prostate Cancer (*n* = 14)	Muscadine Grape Skin (MPX) 4000 mg/day	Positive: Lengthened PSA doubling time (5.3 months increase) in a subset of patients. Negative: Gastrointestinal toxicity at high doses; lack of effect in some genetic subtypes.	[157]
RCT	Metabolic Syndrome (*n* = 74)	150 mg or 1000 mg/day for 16 weeks	Positive: None. Negative: No improvement in inflammatory or metabolic markers; high dose increased total cholesterol.	[158]

## Data Availability

No new data were created or analyzed in this study. As a comprehensive review article, this work synthesizes and discusses findings and data that have been previously published and are available in the scholarly literature. All supporting information and data discussed can be found in the reference list.

## Abbreviations

The following abbreviations are used in this manuscript:

ACC	acetyl-CoA carboxylase
ACLY	ATP citrate lyase
ADMET	absorption, distribution, metabolism, excretion, toxicity
AI	artificial intelligence
Akt	Akt serine/threonine kinase
Apaf-1	apoptotic protease-activating factor 1
Arg-1	arginase-1
ATP	adenosine triphosphate
Bax	Bcl-2-associated X protein
Bcl-2	B-cell lymphoma 2
Bid	BH3-interacting domain death agonist
CAR-NK	chimeric antigen receptor natural killer
CD36	cluster of differentiation 36
c-Myb	v-myb avian myeloblastosis viral oncogene homolog
COX-2	cyclooxygenase-2
CTL	cytotoxic T lymphocyte
ΔΨm	mitochondrial membrane potential
EMT	epithelial–mesenchymal transition
ERK	extracellular signal-regulated kinase
ETC	electron transport chain
FABP5	fatty acid-binding protein 5
FADD	Fas-associated via death domain
FASN	fatty acid synthase
FAOX	fatty acid oxidation
G-MDSC	granulocytic myeloid-derived suppressor cell
GLUT1	glucose transporter type 1
GLUT4	glucose transporter type 4
GPx	glutathione peroxidase
GSK-3β	glycogen synthase kinase 3 beta
HK2	hexokinase 2
HMGB1	high-mobility group box 1
IAPs	inhibitor of apoptosis proteins
IFN-γ	interferon gamma
IκB	inhibitor of kappa B
JAK2	Janus kinase 2
JNK	c-Jun N-terminal kinase
LDHA	lactate dehydrogenase A
MAPK	mitogen-activated protein kinase
MINK1	misshapen-like kinase 1
M-MDSC	monocytic myeloid-derived suppressor cell
mPTP	mitochondrial permeability transition pore
mTOR	mechanistic target of rapamycin
mTORC2	mTOR complex 2
mtROS	mitochondrial reactive oxygen species
NF-κB	nuclear factor kappa-light-chain-enhancer of activated B cells
NHL	non-Hodgkin’s lymphoma
NKG2D	natural killer group 2D
NLRP3	NLR family pyrin domain containing 3
Nrf2	nuclear factor erythroid 2–related factor 2
OXPHOS	oxidative phosphorylation
PD-1	programmed cell death protein 1
PD-L1	programmed death-ligand 1
PGC-1α	peroxisome proliferator-activated receptor gamma coactivator 1-alpha
PI3K	phosphoinositide 3-kinase
PIM-1	proviral integration site for Moloney murine leukemia virus-1
PKM2	pyruvate kinase M2
PLGA	poly(lactic-co-glycolic acid)
PPAR-α	peroxisome proliferator-activated receptor α
PPARβ/δ	peroxisome proliferator-activated receptor β/δ
QSAR	quantitative structure–activity relationship
ROS	reactive oxygen species
SAR	structure–activity relationship
SIRT1	sirtuin 1
SIRT3	sirtuin 3
SOD	superoxide dismutase
STAT3	signal transducer and activator of transcription 3
TAM	tumor-associated macrophage
TGF-β1	transforming growth factor beta 1
TLR4	toll-like receptor 4
TME	tumor microenvironment
TNF-α	tumor necrosis factor alpha
Treg	regulatory T cell
TXNIP	thioredoxin-interacting protein
ULBP2	UL16 binding protein 2
XIAP	X-linked inhibitor of apoptosis protein
